# Long-term outcomes and prognostic markers in gallbladder cancer

**DOI:** 10.1097/MD.0000000000011396

**Published:** 2018-07-13

**Authors:** Xiwei Cui, Sha Zhu, Zhihang Tao, Xinghao Deng, Yexiao Wang, Yuanjing Gao, Yue Liao, Weijun Ma, Yiwen Zhang, Xuelei Ma

**Affiliations:** a Cancer Center, State Key Laboratory of Biotherapy, West China Hospital of Sichuan University; b Department of Oncology, West China Hospital of Sichuan University, Chengdu, Sichuan, China.

**Keywords:** gallbladder cancer, neutrophil–lymphocyte ratio, prognostic factors, survival

## Abstract

Cancer-related inflammation and systemic inflammatory markers have been widely recognized as an essential part in tumor multiplication, invasion, and metastasis of tumor cells. This study aimed to estimate and compare the prognostic value of various biomarkers on overall survival (OS) in patients with gallbladder cancer patients.

We performed a retrospective study of 159 patients received different therapies in West China Hospital from 2009 to 2014. The preoperative biomarker data, including neutrophil–lymphocyte ratio (NLR), platelet–lymphocyte ratio (PLR), monocyte–lymphocyte ratio (MLR), lactate dehydrogenase, and alkaline phosphatase, as well as other clinical information, were obtained from electronic record. And the receiver operating characteristic curves were used to analyze the optimal cut-off values of them. Kaplan–Meier survival analysis and Cox proportional hazard model analysis were applied to evaluate the association between markers and OS.

The optimal cut-off value was 4.39 for NLR, 181.85 for PLR, 0.30 for MLR, and 3.02 for carcinoembryonic antigen (CEA). Kaplan–Meier analysis and univariate Cox analysis both demonstrated the significant prognostic value of NLR, MLR, and CEA. However, PLR failed to be a significant predictor of OS. The multivariate Cox analysis showed that preoperative NLR and CEA were independent prognostic factors for OS.

Advanced tumor/node/metastasis stage, enhanced pretherapeutic NLR, and CEA were significantly associated with worse OS of gallbladder cancer patients. Furthermore, NLR was a better prognostic factor than CEA in advanced T (T3–T4) stage patients, while CEA was better for early T (T1–T2) stage, early N (N0–N1) stage, and early M (M0) stage patients.

## 1 Introduction

Gallbladder cancer (GBCA) is one of the most malignant tumors in biliary system, with a poor overall survival (OS) rate and high degree of malignancy. The overall incidence rate of GBCA was 1.31% (2012), and current studies showed significant increase of incidence rates for GBCA among certain patients.^[1]^ Cholecystectomy is the most common and effective treatment strategy, as it has been combined with adjuvant chemotherapy or adjuvant radiotherapy in several cases.^[2]^ However, some of these inflammatory prognostic factors are still controversial, and have no optimal cut-off values. Therefore, it is of importance to estimate and compare the prognostic value of different new factors for the OS of GBCA patients.

Cancer-related inflammation is a hot research area of recent studies, and a large number of studies have regarded inflammatory markers as an important part in the tumor microenvironment modulation system. The high neutrophil–lymphocyte ratio (NLR) has been proved to be related with the aggressiveness and growth of tumor, which results in poor prognosis in various types of cancer. Moreover, it has been confirmed that platelet is associated with angiogenesis, microvascular permeability, and extravasation of tumor mass.^[3]^ Consequently, NLR and platelet–lymphocyte ratio (PLR) show significant value in prognosticating the outcome of different cancer patients, including lung cancer,^[4]^ liver cancer,^[5]^ colorectal cancer,^[6]^ and prostate cancer.^[7]^ And NLR has been verified as a more powerful prognostic factor in recent studies. Also, monocyte–lymphocyte ratio (MLR), lactate dehydrogenase (LDH), and alkaline phosphatase (ALP) were reported as prognostic biomarkers. The pretherapeutic NLR has been widely applied to measure prognosis of cancer patients, yet the prognostic value of other biomarkers is still controversial.

Based on the above-mentioned situation, we initiated our study aiming to estimate the prognostic value of various clinical factors and inflammatory biomarkers, according to the median survival time (MST) and OS of GBCA patients with different treatment. Therefore, we performed a retrospective analysis on a cohort of 159 appropriate GBCA cases in a single cancer center.

## 2 Methods

### 2.1 Patients and data collection

We screened against criteria of exclusion including suspected cases and lose of follow-up information, and 560 patients with GBCA who received treatment in West China Hospital (Sichuan China) from September 2009 to August 2017 were retrospectively reviewed. One hundred fifty-nine patients who received treatment before December 2014 were enrolled in this retrospective study, according to accurate pathological diagnoses. Patients included must be older than 18 years old and have neither infection, nor any kind of therapies or diseases that could influence the concentration of related biomarkers, including hematological system disease, chronic or acute inflammation in other organs, and GBCA complicated by other kind of cancers. In addition, the clinical data of patients should be complete.

All clinical characteristics of patients were extracted from electronic records. The tumor/node/metastasis (TNM) stages of patients were estimated based on pathological and iconography reports according to the American Joint Committee on Cancer (7th edition). Clinical information composed of imaging examination results such as abdominal ultrasound, computed tomography (CT), and magnetic resonance (MR) scan, and serological tumor markers, including carbohydrate antigen 19-9, carcinoembryonic antigen (CEA), LDH, and ALP, and other inflammatory markers including NLR, PLR, and MLR. The pathological types and GBCA diagnoses were examined based on the defining criteria defined by World Health Organization (2010 edition).

This study had already obtained necessary approval from the institution ethics commission of Sichuan University, and the patients refused to participate had been excluded, all the informed consents of included cases had been obtained before the research.

### 2.2 Follow up

The vast majority of our patients had been discharged home, they were required to receive examinations every 6 months or at least 3 years at outpatient clinics to monitor the evolvement or recurrence of tumor, and the detecting methods were mainly CT, MR, and ultrasonography. The median follow-up period was 8.06 months (ranged from 0.23 to 74.3 months). The OS time was calculated as the interval from the first received resection surgery or specific treatment to that of death or the last follow up. And the follow-up data were obtained via outpatient clinic visits and phone calls. Moreover, the surviving cases with no cancer progression were eliminated in the last round of follow-up.

### 2.3 Statistical analyses

All the statistical analyses were performed using the Statistical Package for the Social Sciences for Windows version 21.0 software program. Clinical data were presented as the mean ± standard deviation, and enumeration data were expressed by percentage, and compared by the chi-squared test. Kaplan–Meier survival curves and log-rank analysis were performed to estimate the survival curves and compare the differences between them. The independent factors associated with patients’ prognoses were analyzed by Cox proportional hazards model, and only the covariables showed significant relativity in univariate analyses were included into multivariate Cox analysis. Hazard ratios (HRs) were presented as relative risks with corresponding 95% confidence intervals (CIs). All the tests were 2-sided, and *P* value < .05 was considered statistically significant.

## 3 Results

### 3.1 Base-line characteristics of patients

Among all the 159 GBCA patients included, there were 51 males (32.1%) and 108 females (67.9%), with a ratio of 0.472 (male/female). The median age at diagnosis was 64 (range 40–96). Ninety patients (56.6%) accepted radical cholecystectomy operation with all the tumor mass and regional lymph nodes cleared, and the rest 69 patients (43.4%) received different kind of treatments including chemotherapy, radiotherapy, intervention surgery, and palliative care. Pretherapeutic jaundice was performed in 47 patients (29.6%). And liver involvement was found in 97 patients (61.0%). There were more patients with liver involvement in high NLR group (*P* < .001) and MLR group (*P* < .001). NLR, MLR, and CEA were also significantly associated with gender, PLR, CA19-9, LDH, and ALP. MLR and CEA were also significantly associated with liver involvement. Detail information could be accessed in Table 1.

**Table 1:**
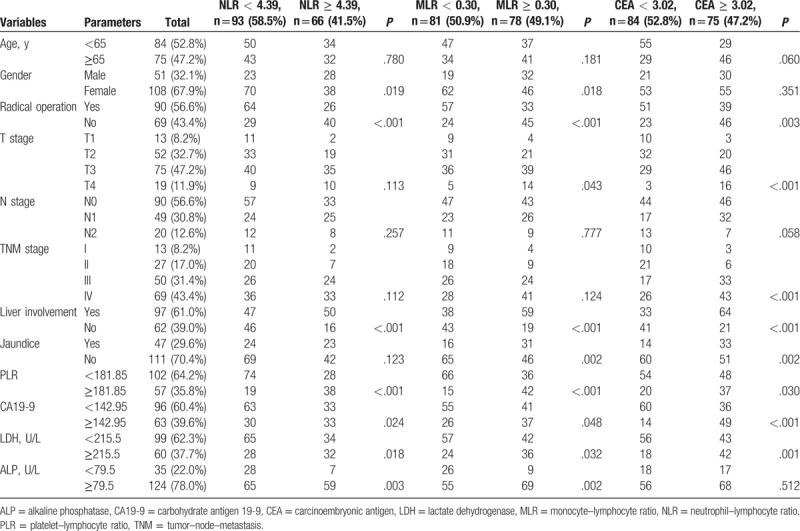
Baseline characteristics of patients with gallbladder cancer.

### 3.2 OS according to clinical factors

Among the cases involved, 137 death cases made up for 86.2% of the entire cohort (N = 159) while the rest 22 survival cases got a proportion of 13.8%. The median survival time was 8.067 months while the OS of estimated cumulative 6-month, 1-year, and 3-year survivals for this patient population was 62.9 ± 3.8%, 37.7 ± 3.8%, and 18.5 ± 3.1%, respectively. Ninety-seven cases were found liver involvement (61.0%). The MST for patients with liver involvement was 7.23 months, and the 3-year survival time was 11.2 ± 3.2%, while those without liver involvement got an MST of 11.77 months and a 3-year OS rate of 30 ± 5.9%. And liver involvement was verified significantly associated with a worse outcome (*P* < .001), according to the univariate analysis showed in Table 2.

**Table 2:**
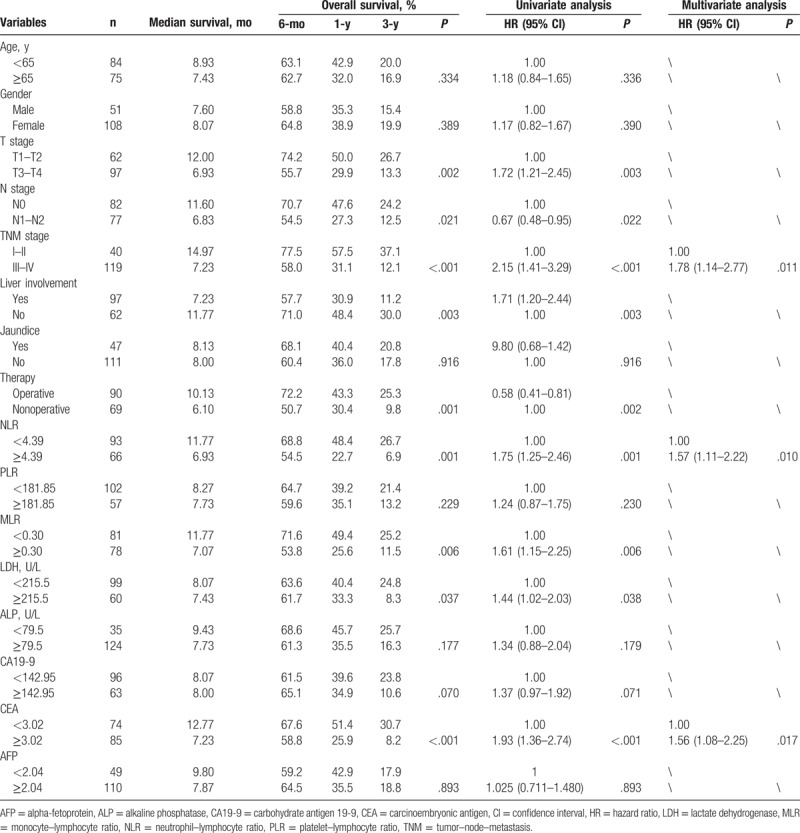
Univariate and multivariate analyses of factors associated with overall survival in patients with gallbladder cancer.

The results (Table 2) also showed a significant difference between operative group and nonoperative group in MST and OS of patients. The MST and 3-year OS rate of operative group (n = 90) were much higher than those of nonoperative group (n = 69), and the association between operation and OS of patients was significant (*P* = .002, Table 2). However, liver involvement and operation both failed to show significant prognostic value in multivariate Cox regression analysis, while TNM stage was recognized as an independent prognostic factor for OS of GBCA patients, with a multivariate HR of 1.78 and a 95% CI of 1.14 to 2.77 (*P* = .011).

### 3.3 Cut offs of biomarkers

To avoid a predetermined cut-off point, we re-determined the cut offs of CA19-9, CEA, and alpha-fetoprotein (AFP) to measure prognoses instead of using the international diagnose standard. Applying receiver operating characteristic (ROC) curve analysis, we determined the optimal cut-off values of pretherapeutic CA19-9, CEA, AFP, LDH, and ALP according to the maximum joint specificity and sensitivity. The area under the curve of pretherapeutic CA19-9, CEA, AFP, LDH, and ALP, in sequences, were 0.676, 0.715, 0.569, 0.621, and 0.548, and the cut offs were 142.95, 3.02, 2.035, 215.5, and 79.5, respectively. ROC analyses were also applied to NLR, PLR, and MLR values. According to the ROC analysis, the area under the ROC curves was 0.592, 0.516, and 0.609, respectively. And the optimal cut offs were 4.39, 181.85, and 0.30, respectively. All the cut offs above were defined with both maximum specificity and sensitivity. In addition, serum bilirubin level exceeding 34.2 μmol/L (2 mg/dL) was defined as jaundice.

### 3.4 OS according to serum and inflammation biomarkers

**Figure 1:**
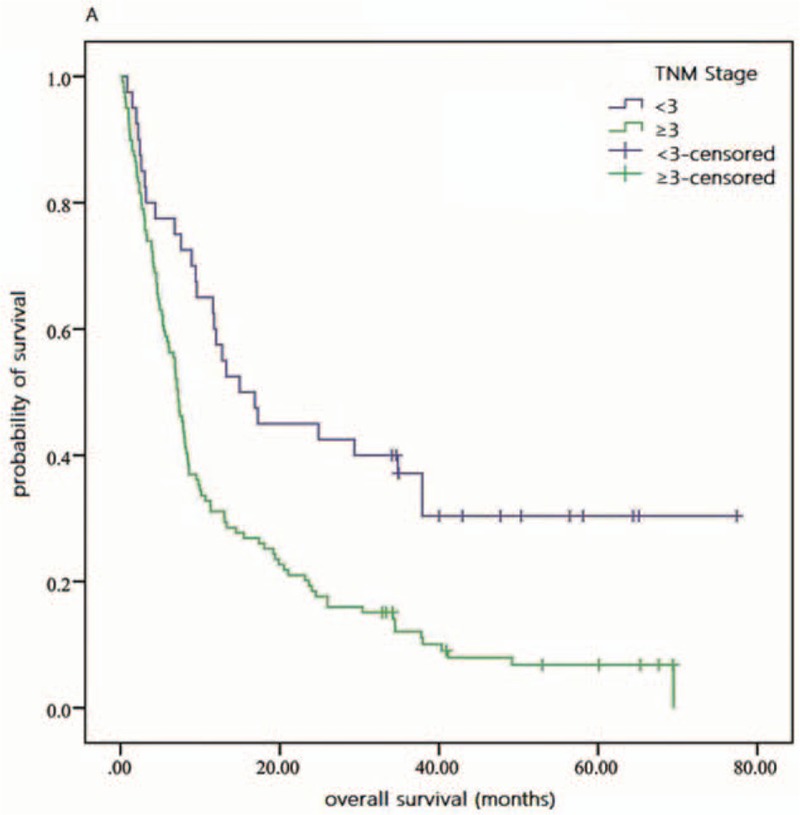
Overall survival of early and advanced TNM stages patients. TNM = tumor–node–metastasis.

**Figure 2:**
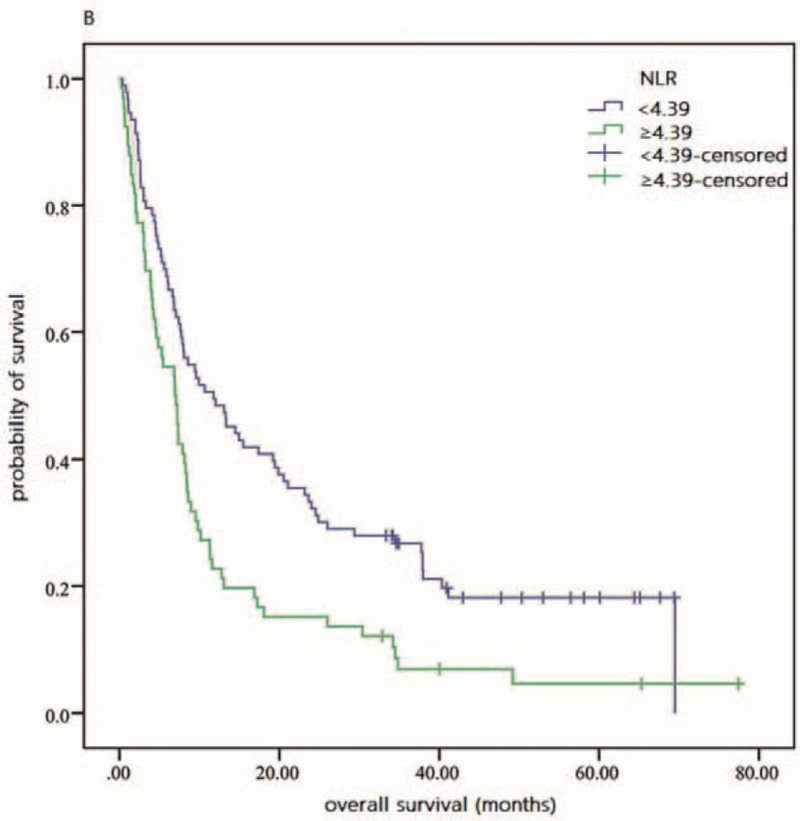
Overall survival of patients with different NLR values. NLR = neutrophil–lymphocyte ratio.

**Figure 3:**
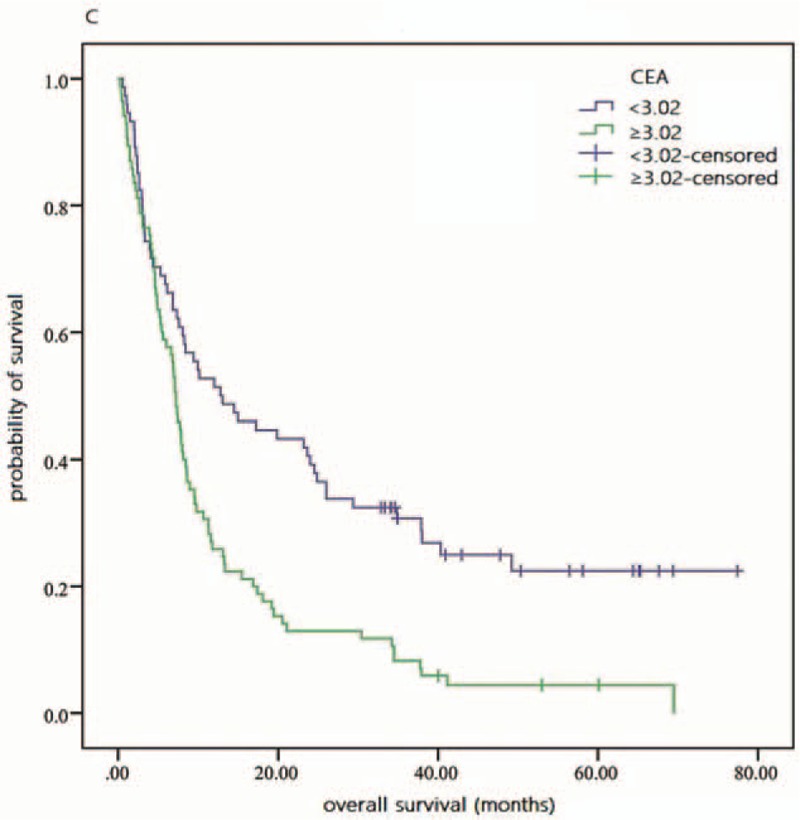
Overall survival of patients with different CEA values. CEA = carcinoembryonic antigen.

As shown in Fig. 1, by the last round of follow up, the accumulative total deaths of high and low NLR groups were 62 and 68, respectively. Based on Table 2 and Fig. 2, the 3-year OS rate of high and low NLR groups was 6.9 and 26.7 in Kaplan–Meier analysis, respectively, and statistically significant difference was verified in log-rank test of 2 groups (*P* = .001). And the univariate HR and 95% CI of NLR were 1.75 and 1.25 to 2.46 (*P* = .001), respectively, according to Cox regression analysis. MLR, CA19-9, LDH, and CEA also showed significant prognostic value for patients’ OS in univariate analysis (Table 2 and Fig. 3). In addition, several clinical parameters including advanced TNM stages, liver involvement, and nonoperation were found associated with worse prognosis in univariate analysis.

However, in multivariate analysis (Cox regression analysis method: forward: likelihood ratio), only NLR, CEA, and TNM stage were verified as independent prognostic markers for OS of GBCA patients. And, the multivariate HR was 1.57, 1.56, and 1.78, the 95% CI was 1.11 to 2.22 (*P* = .010), 1.08 to 2.25 (*P* = .017), and 1.14 to 2.77 (*P* = .011), respectively. And among the subgroups, NLR was a better prognostic factor in advanced T stage patients (*P* = .007), while CEA was better in early T stage (*P* = .001), early N stage (*P* = .007), and early M stage (*P* = .004) patients (Table 3). All the multivariate analysis of HRs mentioned in subgroup approximately 2, which indicates statistically significance (Tables 2 and 4).

**Table 3:**
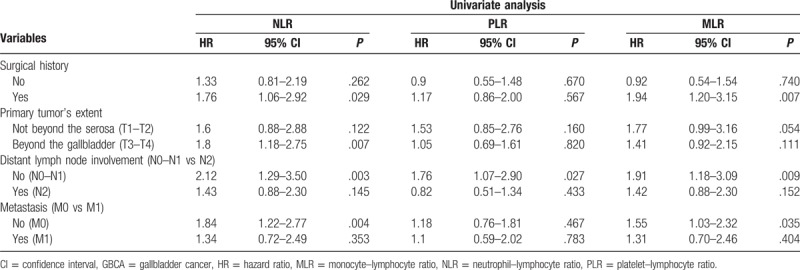
Univariate analysis of factors associated with overall survival in GBCA patients in different subgroups.

**Table 4:**
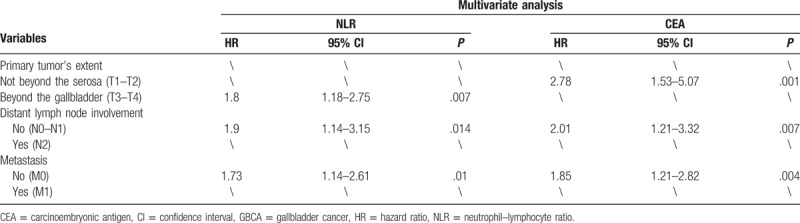
Multivariate analysis of factors associated with overall survival in GBCA patients in different subgroups.

## 4 Discussion

Recently, numerous biomarkers were found valuable in predicting prognosis of cancer patients. Among them, NLR, PLR, and MLR were certified to be closely associated with inflammation and immunity status in cancer patients, thus being widely used in similar studies to estimate patients’ outcomes. And in our study, NLR and CEA also showed high power of test in independent prognosis. In addition, we determined the optimal cut-off values of these factors by ROC curve analysis while there are no consistent cut-off values in preexisting studies.

NLR has been reported as an independent prognostic factor for OS of cancer patients, corresponding to our conclusion. In terms of the mechanism, the function of different types of cells in tumor pathologic process and related inflammation is the key point. The neutrophil, known as a kind of inflammation cell, influences tumor initiation and progression in the tumor microenvironment.^[8]^ It plays an important role in the proliferation and regenesis of tumor cells, and could also promote angiogenesis and metastasis, interfere adaptive immune responses, and influence responses to hormones and chemotherapeutic medicament.^[9]^ The neutrophil also enhances the bioavailability and bioactivity of vascular endothelial growth factor (VEGF),^[10]^ which is indispensable for the angiogenesis in tumor development, recrudesce, invasion, and metastasis.^[11]^ Meanwhile, lymphocyte was also reported as an significant role of antitumor immunity in several clinical and experimental studies.^[12]^ The enhancement of NLR always indicates the increase of neutrophil amount or the decrease of lymphocyte count, which might be caused by malignant tumors. And tumor cells can disturb the inflammatory route and immune response system, in order to create a more convenient microenvironment for tumor growth and metastasis. This may explain why patients with higher NLR got worse outcome.^[13]^

PLR is another factor of cancer-related inflammation, which can always indicate the increase of platelet count. And, it has been demonstrated that platelet plays an important role in tumor growth and metastasis in many studies. However, the pathological mechanisms are still controversial. The mainstream opinion is that the growth of tumor cell will activate thrombopoietin by releasing certain inflammatory factors, then, PDGF and tumor necrosis factor (TNF) delivered by increased platelets can accelerate the growth of tumor cell.^[14]^ Platelet can also promote the invasion and recurrence of tumor cell, and has been recognized as one of the main sources of cytokines including VEGF and transforming growth factor β (TGF-β), which have significant influence in tumor angiogenesis.^[15]^ In addition, MLR was ever confirmed as an independent prognostic biomarker for cancer patients’ OS in previous studies.^[16]^ Monocyte has been widely approved as an significant role in immune system. Recent studies showed that monocyte, especially Patrolling Monocyte, could also participate the antitumor procedure.^[17]^ Monocyte can patrol in blood vessels and clean the cell debris in the micro environment.^[18]^ By raising natural killer T cells and releasing TNF-α, IL-12, and nitric oxide,^[19]^ monocyte can restrain the growth and recurrence of tumor cells. It has been proved that immune cells can accelerate tumor metastasis by more and more experimental data. By raising immunosuppressive cells, tumor cells can get away from the attack of killer cells. However, certain type of monocyte can prohibit the metastasis of tumor.^[20]^

As for the clinical factors involved, according to Table 1, radical operation was still the best treatment for GBCA patients, with an HR of 0.58 and the 95% CI was 0.41 to 0.81 (*P* = .002). Patients gender was shown to be significantly associated with NLR and MLR. Race difference among GBCA patients was proved in several researches,^[1]^ but was not reached in this study. Analysis showed significant association between advanced T stage and higher NLR, MLR, and CEA value, that might be the result of the tumor invasion assisted by neutrophil and the antitumor immune procedure of monocyte. We also found the patients with jaundice got higher MLR and CEA, the mechanism remains open for interpretation. Furthermore, among the factors mentioned, NLR value and TNM stage have already been widely recognized as independent prognostic factor for cancer patients. However, CEA have seldom been mentioned by recent studies, but showed significant independent prognostic value for GBCA patients’ OS in multivariate Cox regression analysis.

In addition, we found several deficiencies in our study. Above all, although the sample capacity was larger than those of certain studies, further large sample investigations were still needed to accomplish a more reliable test. Secondly, all present research, including this one, are retrospective, more multicenter and prospective studies are needed to support present theories. Besides, patients included were all Chinese, clinical differences between races were not reached.

## 5 Conclusion

In conclusion, we retrospected 159 GBCA patients who have received different treatment, and it was confirmed that advanced TNM stages and high pretherapeutic NLR and CEA were independent prognostic factors for worse OS of GBCA patients receiving various treatment. Furthermore, NLR and CEA were independent prognostic biomarkers for indicating OS outcome. And, our results suggested that the biomarkers we tested are new and valid indicators besides ordinary examination and statistical analyses. However, it still needs to be verified by further multicenter and large-scale prospective studies. Experimental studies are also needed to excavate more pathological details in tumor inflammatory procedure.

## Author contributions

**Conceptualization:** Sha Zhu, Yexiao Wang, Weijun Ma.

**Data curation:** Xinghao Deng.

**Formal analysis:** Xiwei Cui.

**Funding acquisition:** Sha Zhu.

**Investigation:** Xiwei Cui, Xinghao Deng, Yuanjing Gao, Yue Liao.

**Methodology:** Zhihang Tao, Yiwen Zhang.

**Visualization:** Zhihang Tao.

**Writing – original draft:** Xiwei Cui.

**Writing – review and editing:** Sha Zhu, Yiwen Zhang, Xuelei Ma.
